# Screen Printed Pb_3_O_4_ Films and Their Application to Photoresponsive and Photoelectrochemical Devices

**DOI:** 10.3390/ma11071189

**Published:** 2018-07-11

**Authors:** Riccardo Panetta, Simone Quaranta, Alessandro Latini

**Affiliations:** 1 Dipartimento di Chimica, Università degli Studi di Roma “La Sapienza”, Piazzale Aldo Moro, 5, 00185 Roma, Italy; riccardo.panetta@uniroma1.it; 2 Dipartimento di Ingegneria dell’Informazione, Elettronica e Telecomunicazioni, Università degli Studi di Roma “La Sapienza”, Via Eudossiana, 18, 00184 Roma, Italy; simone.quaranta@uniroma1.it

**Keywords:** Pb_3_O_4_, screen printing, p-n heterojunction, photoresponsive device, photoelectrochemical cell

## Abstract

A new and simple procedure for the deposition of lead (II, IV) oxide films by screen printing was developed. In contrast to conventional electrochemical methods, films can be also deposited on non-conductive substrates without any specific dimensional restriction, being the only requirement the thermal stability of the substrate in air up to 500 °C to allow for the calcination of the screen printing paste and sintering of the film. In this study, films were exploited for the preparation of both photoresponsive devices and photoelectrochemical cell photoanodes. In both cases, screen printing was performed on FTO (Fluorine-Tin Oxide glass) substrates. The photoresponsive devices were tested with I-V curves in dark and under simulated solar light with different irradiation levels. Responses were evaluated at different voltage biases and under light pulses of different durations. Photoelectrochemical cells were tested by current density–voltage (J-V) curves under air mass (AM) 1.5 G illumination, incident photon-to-current efficiency (IPCE) measurements, and electrochemical impedance spectroscopy.

## 1. Introduction

Pb_3_O_4_, commonly known as “minium” or “red lead”, is perhaps the best known lead oxide. Pb_3_O_4_ has been employed since ancient times as a pigment because of its beautiful bright red-orange color. It was used in ancient Rome as a low-cost alternative to vermillion (cinnabar, HgS) as well as in medieval Europe for artistic purposes (e.g., decorative miniatures in manuscripts [1]). Until recently, it was also extensively utilized in anti-corrosive paint formulations. Nevertheless, this application has been phased out because of concerns over lead toxicity, particularly in terms of its environmental impact. Red lead also has been proposed for other uses, such as solar batteries [2] and photocatalysts [3]. Despite the aforementioned problems, Pb_3_O_4_ remains an interesting material with potential intriguing applications, especially because of its optical properties [3]. For this reason, we investigated the properties of lead (II, IV) oxide as n-type semiconductor [4] in photoresponsive and photoelectrochemical thin film devices. Few studies regarding the syntheses of Pb_3_O_4_ nanomaterials are available [5,6,7]. In addition, for thin films, deposition processes are limited to the electrochemical depositions of PbO_2_, which are followed by thermal treatments [4,6,8]. In this work, a new procedure for the deposition of lead (II, IV) oxide films by screen printing is presented as a cheaper and more flexible alternative to existing electrochemical methods, which are limited to electrically conductive substrates. Easily manufactured photoresponsive devices were prepared by printing Pb_3_O_4_ films onto FTO substrates and using CuSCN as a p-type semiconductor [9,10]. Furthermore, films prepared with the same procedure were tested as photoanodes in photoelectrochemical cells using an iodide/triiodide-based electrolyte (commonly employed in dye-sensitized solar cells) and a platinum film on an FTO glass slide as the counter electrode. The photoresponsive devices were tested with I-V curves in dark and under simulated solar light at different irradiation levels. Additionally, responses to different voltage biases and to light pulses of different lengths were also evaluated. The photoelectrochemical cells were tested by current density–voltage (J-V) curves under air mass (AM) 1.5 G illumination, incident photon-to-current efficiency (IPCE) measurements, and electrochemical impedance spectroscopy.

## 2. Experimental

### 2.1. Materials

Lead (II) nitrate (99.0% min; Alfa Aesar, Karlsruhe, Germany) was used for the synthesis of Pb_3_O_4_; acetic acid (99–100%; Sigma Aldrich, Milan, Italy), 5–15 mPa·s ethyl cellulose (48.0–49.5% *w*/*w* ethoxyl basis; Sigma Aldrich), 30–70 mPa·s ethyl cellulose (48.0–49.5% *w*/*w* ethoxyl basis, Sigma Aldrich), ethanol absolute (VWR chemicals, Milan, Italy); anhydrous terpineol (Sigma Aldrich) were used for screen printing paste preparation; 3 mm thick FTO glass slides with sheet resistances of 10 Ω (XOP Fisica, Castellon, Spain) were used as substrates for film depositions; zinc powder (6–9 micron, 97.5%, Alfa Aesar) and hydrochloric acid (≥37%, Sigma Aldrich) were used for FTO etching; copper (I) thiocyanate (96%, Alfa Aesar) and n-propyl sulfide (99%, Alfa Aesar) were used for p-type semiconductor film deposition; silver conductive paint (RS Components, Cinisello Balsamo, Italy) was used for the electrical contacts on p-type semiconductor; hydrogen hexachloroplatinate (IV) hydrate (40% Pt by weight, Chempur, Karlsruhe, Germany) was used for the deposition of the catalytic film on the cathode of photoelectrochemical cells; EL-HPE high performance electrolyte (Sigma Aldrich) was used as electrolyte in photoelectrochemical cells.

### 2.2. Methods

#### 2.2.1. Pb_3_O_4_ Powder Synthesis and Paste Preparation

Lead (II, IV) oxide powder was produced by calcination at 465 °C for 3 h in air of lead (II) nitrate. The obtained Pb_3_O_4_ powder was used to prepare the screen printing paste according to the procedures developed by Ito et al. [11] on an equal oxide volume basis.

#### 2.2.2. Screen Printing of Pb_3_O_4_ Films

The glass slides used for screen printing of Pb_3_O_4_ films for photoresponsive devices were prepared as follows. Part of the FTO area (square glass slides, 2.5 × 2.5 cm^2^) was treated with zinc powder and hydrochloric acid to form an electrically insulating zone to prevent short circuit as well as to allow for photoinduced charge separation and collection after the deposition of copper (I) thiocyanate (see Section 2.2.3). Accordingly, two FTO distinct areas were fabricated on the same side of the glass: one for the electron contact and the other for the hole contact. Before deposition of the semiconductor film, the glass slides were ultrasonicated for 20 min in ethanol, then in a 1% Liquinox solution in deionized water for 20 min, followed by rinsing with deionized water and two more ultrasonication processes in Milli-Q water (5 min each). The square films of Pb_3_O_4_ (about 1 × 1 cm^2^) were prepared by screen printing ethyl cellulose-based pastes in terpineol by using a manual screen printing table (model 60–90, Mismatic, Fizzonasco, Italy) equipped with a 34T polyester mesh screen. The printing process was repeated to obtain a final film thickness of about 5 μm. Photoanodes’ thickness was measured by means of a stylus profilometer (model MAP3D-25, A.P.E. Research, Basovizza, Italy, nominal resolution 10 nm). Pb_3_O_4_ films were deposited on the first FTO area and on the insulating zone. After printing, the films were gradually heated in air at 325 °C for 5 min, at 375 for 5 min, at 450 °C for 15 min, at 500 °C for 15 min, and finally at 465 °C for 12 h. The last step (465 °C for 12 h) represents a key point for the preparation of Pb_3_O_4_ films; it is instrumental to regulate the correct oxygen content after the reduction of Pb_3_O_4_ by the organic material present in the paste. Pb_3_O_4_ films with the same thickness as above for the photoelectrochemical cells (about 0.32 × 0.32 cm^2^) were deposited on glass slides with a single FTO-covered area.

#### 2.2.3. Photoresponsive Devices and Photoelectrochemical Cells Preparation

**Figure 1:**
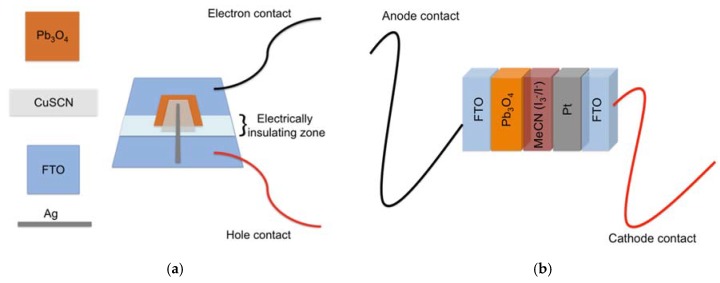
(**a**) A schematic representation of an assembled photodiode; (**b**) A schematic representation of an assembled photoelectrochemical cell.

A CuSCN film was deposited over the Pb_3_O_4_ film from a saturated solution in n-propyl sulfide according to the procedure described by Kumara et al. [12]. To avoid a short circuit of the device, the CuSCN film area should not touch the FTO layer underneath the Pb_3_O_4_ film. Thus, the CuSCN layer was stacked on the Pb_3_O_4_ film and the electrically insulating zone. Finally, the hole contact was fabricated using silver conductive paint for connecting the hole transport material (CuSCN) with the second FTO area. A schematic representation of the assembled device is shown in Figure 1a.

The photoelectrochemical cells were fabricated following the same procedure used for the assembling DSSCs (Dye-Sensitized Solar Cells) as reported in our previous works [13,14,15]. A schematic representation of the assembled device is shown in Figure 1b.

### 2.3. Structural and Morphological Characterization

#### 2.3.1. XRD

Diffraction analysis of the Pb_3_O_4_ and CuSCN films was performed using a Panalytical X’Pert Pro MPD diffractometer (Cu Kα radiation, λ = 0.154184 nm) (Panalytical, Almelo, Netherlands) equipped with a thin film attachment, in low incident beam angle mode (0.5° in 2θ) to minimize the substrate contribution to the diffraction pattern. The angular range used was 10–90° (in 2θ).

#### 2.3.2. UV-Vis Spectroscopy

Diffuse reflectance UV-Vis spectroscopy was used to measure the band gap of the Pb_3_O_4_ films. Measurements were performed with a UV2600 UV-Vis (Shimadzu, Kyoto, Japan) spectrophotometer equipped with a ISR-2600 Plus integrating sphere. The gap values were calculated by determining the absorption edge using the Kubelka-Munk function. Furthermore, the transition nature was determined by comparing the Kubelka-Munk function’s absorption edges with Tauc’s plots using different exponents [16].

#### 2.3.3. FESEM

Field emission scanning electron microscopy analysis of the films’ morphology and 3D CuSCN-Pb_3_O_4_ p-n heterojunction were performed by means of a Auriga FESEM (Zeiss, Oberkochen, Germany). Furthermore, EDS analysis (Bruker AXS, Karlsruhe, Germany) of the heterojunction was performed. The FESEM apparatus is equipped with a Schottky field emission Gemini electron column. Operating range 100 V–30 kV. Resolution: 1.0 nm at 15 kV.

### 2.4. Device Test

#### 2.4.1. Photoresponsive Device Test

##### I-V Curves and Response of the Device

I-V characteristics were acquired by using a Solartron Analytical 1286 electrochemical interface (EI) (Solartron Analytical, Leicester, England), under simulated solar light at different irradiation levels, over the range 334–1036 W m^−2^ and in dark. The light was generated by an Asahi Spectra (Tokyo, Japan) HAL-320 class A solar simulator, and a calibrated Asahi Spectra Sun Checker was used to check the intensity of the simulated solar radiation.

A Solartron Analytical 1286 electrochemical interface (EI) and a white LED, coupled with a programmable function generator AMEL model 568 (AMEL, Milan, Italy), were used to evaluate the current response of the photoresponsive device using forward-biased voltage pulses at constant irradiance as well as using light pulses at constant forward bias.

#### 2.4.2. Photoelectrochemical Cell Test

##### J-V Curves

J-V curves, under simulated AM 1.5 G solar radiation, were collected with the Solartron Analytical 1286 electrochemical interface (EI).

##### Electrochemical Impedance Spectra

Electrochemical impedance spectra (EIS) under illumination with a DC bias corresponding to the maximum power point of the photovoltaic device were acquired by using the EI coupled with the Solartron Analytical 1260 frequency response analyzer (FRA).

##### Incident Photon to Current Conversion Efficiency

The incident photon to current conversion efficiency (IPCE) curves were recorded in DC mode using a custom-made apparatus controlled by a LabVIEW-based software (version 2010, National Instruments, Austin, TX, USA) [13,14,15].

## 3. Results and Discussion

### 3.1. XRD

**Figure 2:**
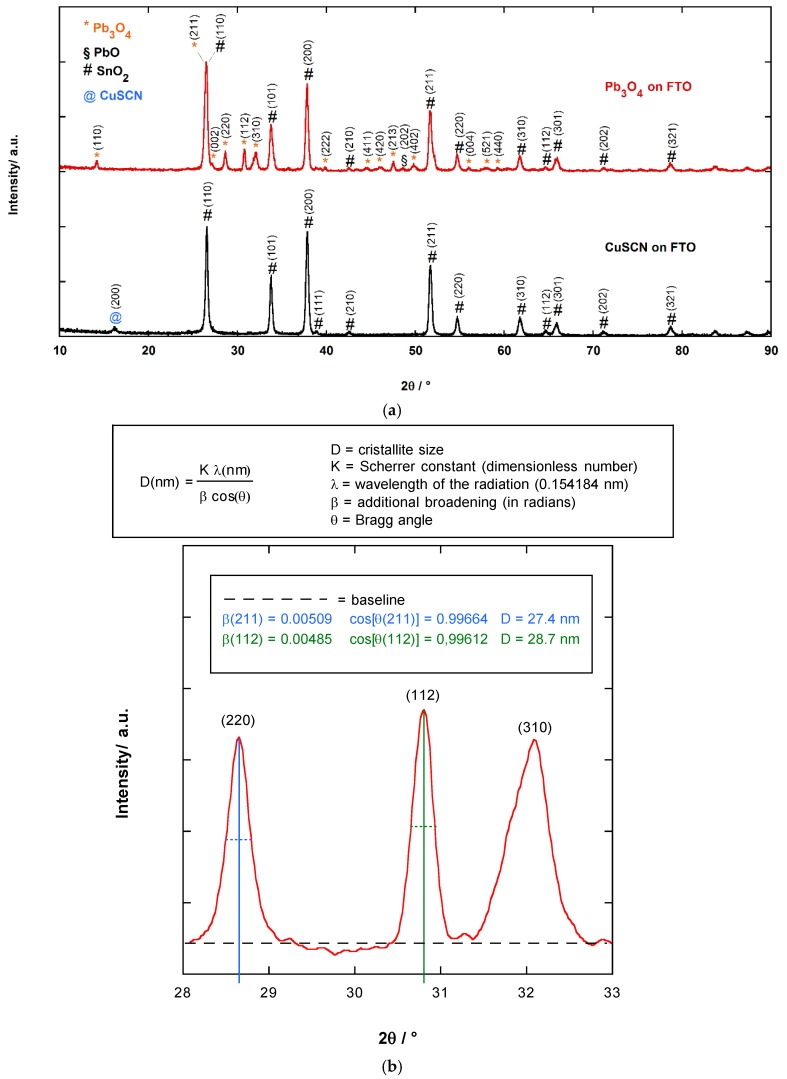
(**a**) XRD patterns of multilayer made of a Pb_3_O_4_ film and CuSCN film, both deposited on FTO; (**b**) Estimation of the mean crystallite size in the Pb_3_O_4_ film by Scherrer equation.

The XRD patterns of Pb_3_O_4_ film and CuSCN film, both deposited on FTO, are reported in Figure 2a. The observed peaks in the experimental patterns were attributed to the following phases: Pb_3_O_4_ (ICDD collection code: 03-6253), CuSCN (ICDD collection code: 00-0124), and SnO_2_ (ICDD collection code: 03-9176) [17]. The main contribution of Pb_3_O_4_ film to the diffraction pattern stems from the (211) reflection (2θ = 26.45°). This peak was overlapped to the (110) peak of SnO_2_. Thus, to estimate the mean size of Pb_3_O_4_ crystallites film through the Scherrer equation [18], (220) (2θ = 28.65°) and (112) (2θ = 30.81°) reflections were taken into account. The average crystallites size, *d*, turned out to be of 28 ± 1 nm (see Figure 2b). The same calculation could not be performed on the CuSCN film because the intensity of its main reflection (200) (2θ = 16.19) was too low.

### 3.2. UV-Vis Spectroscopy

**Figure 3:**
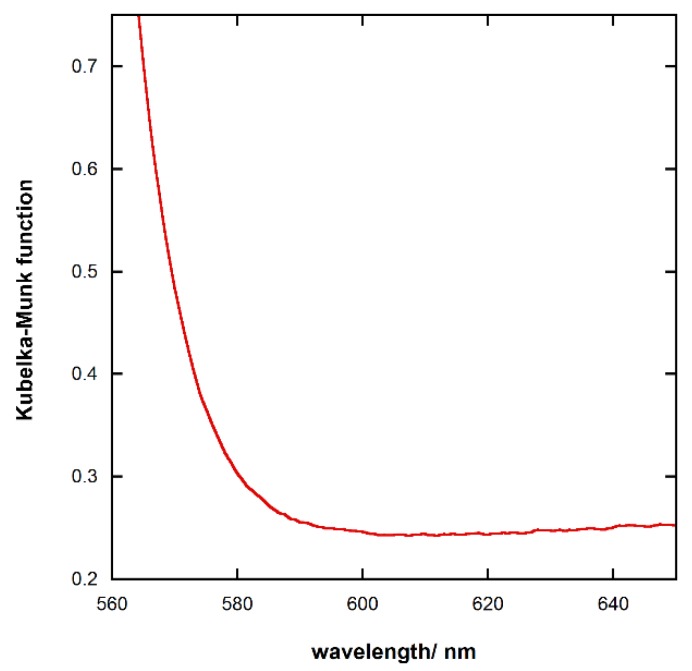
UV-Vis spectrum of Pb_3_O_4_.

Pb_3_O_4_ film band gap was found to be 2.14 eV. This value was attained by converting the reflectance spectra into pseudoabsorbance by means of the Kubelka-Munk function and using the wavelength of the absorption onset (see Figure 3). The gap value was in good agreement with the 2.12 eV reported by Sharon et al. [4]. Furthermore, the nature of the band gap transition was determined by a comparison of the values obtained varying the exponent in the Tauc’s plots with that from the absorption onset. As shown in Table 1, the transition results to be allowed indirect.

### 3.3. FESEM

**Figure 4:**
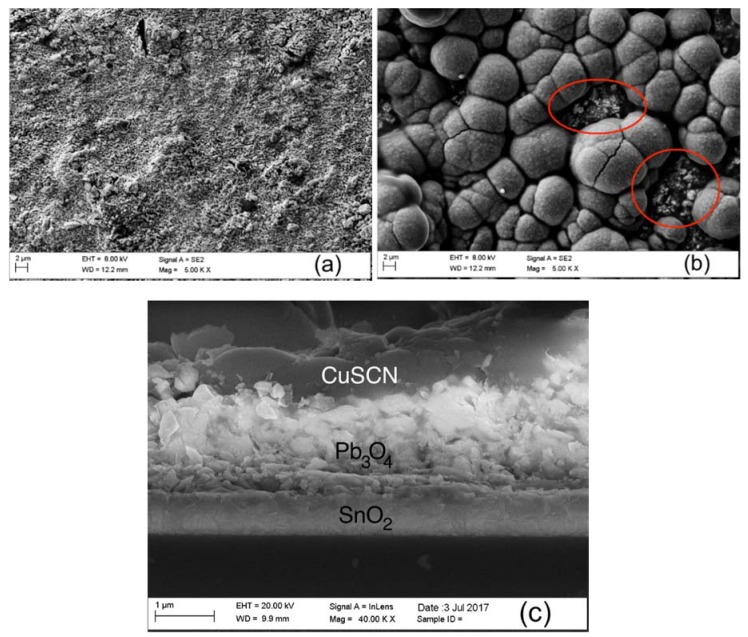
(**a**) SEM image of the Pb_3_O_4_ film; (**b**) SEM image of CuSCN film; (**c**) SEM image of a cross section of the heterojunction.

**Figure 5:**
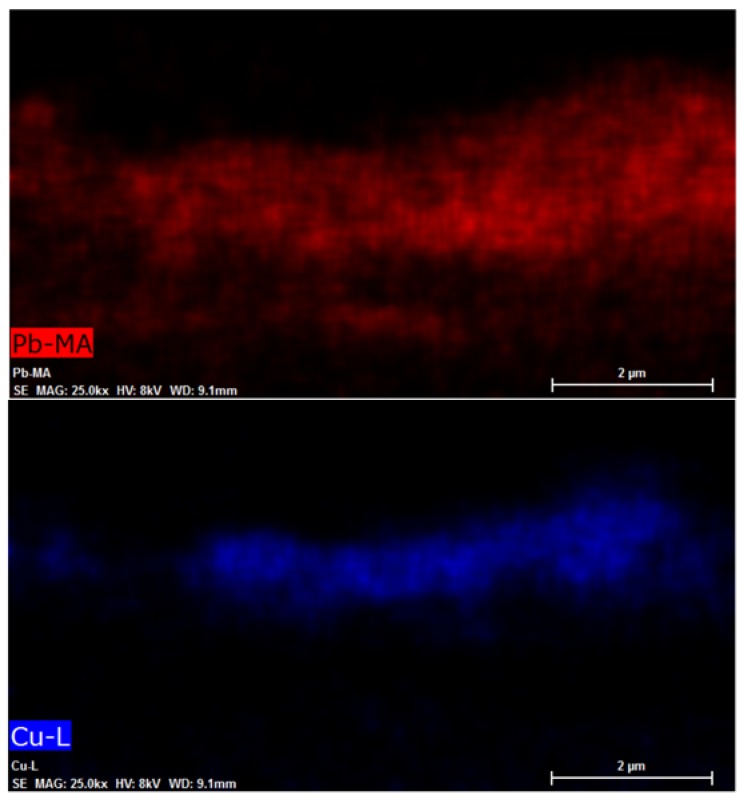

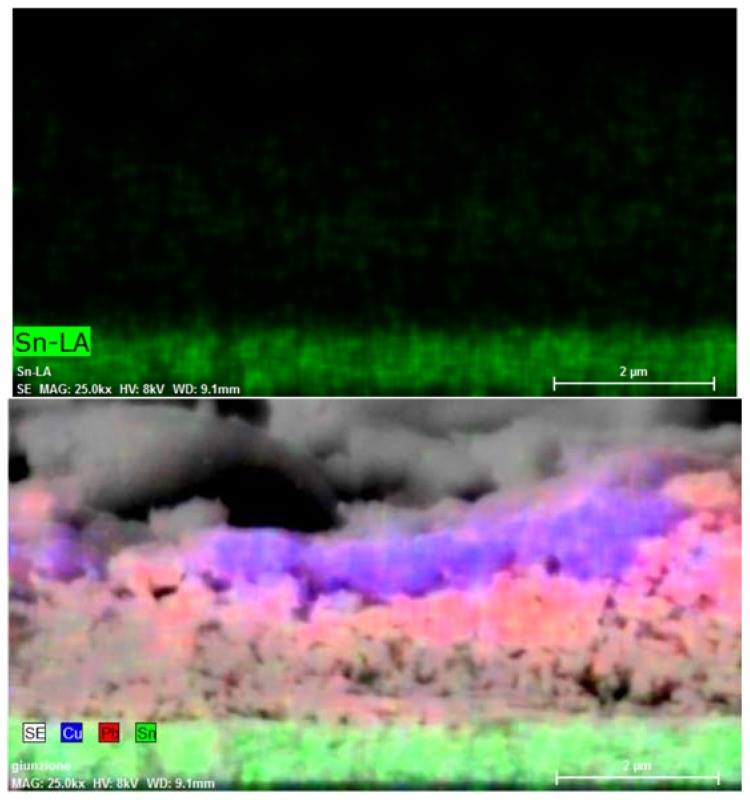
EDS microanalysis maps of the heterojunction.

The morphology of the films and 3D CuSCN-Pb_3_O_4_ p-n heterojunction was analyzed by FESEM. The images (Figure 4) show that the Pb_3_O_4_ film has a porous, homogenous texture (Figure 4a) and that the CuSCN film does not evenly cover the Pb_3_O_4_ film underneath, as highlighted by the red circles in Figure 4b. Finally, the image in Figure 4c illustrates a section of the heterojunction. The two films can be distinguished by the high atomic number of lead, which makes the Pb_3_O_4_ film brighter than the CuSCN and FTO films. Furthermore, the EDS microanalysis map of the heterojunction is shown in Figure 5. These images demonstrate a compenetration of the two layers and, consequently, the formation of a 3D heterojunction between CuSCN and Pb_3_O_4_.

### 3.4. I-V Curves of Photoresponsive Device

**Figure 6:**
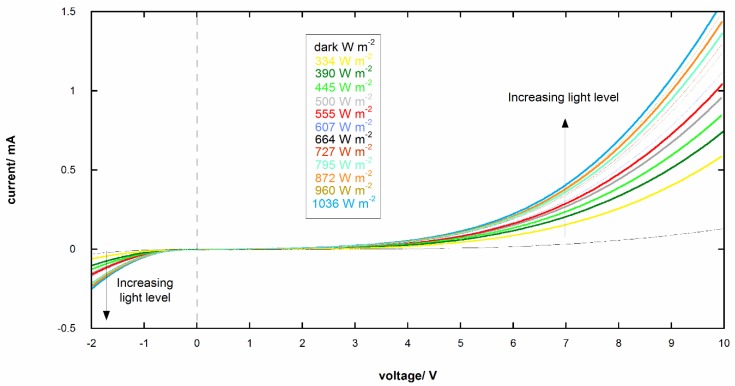
I-V curves of the photoresponsive device under simulated solar light with different irradiation levels in the range 334–1036 W m^−2^ and in dark.

**Figure 7:**
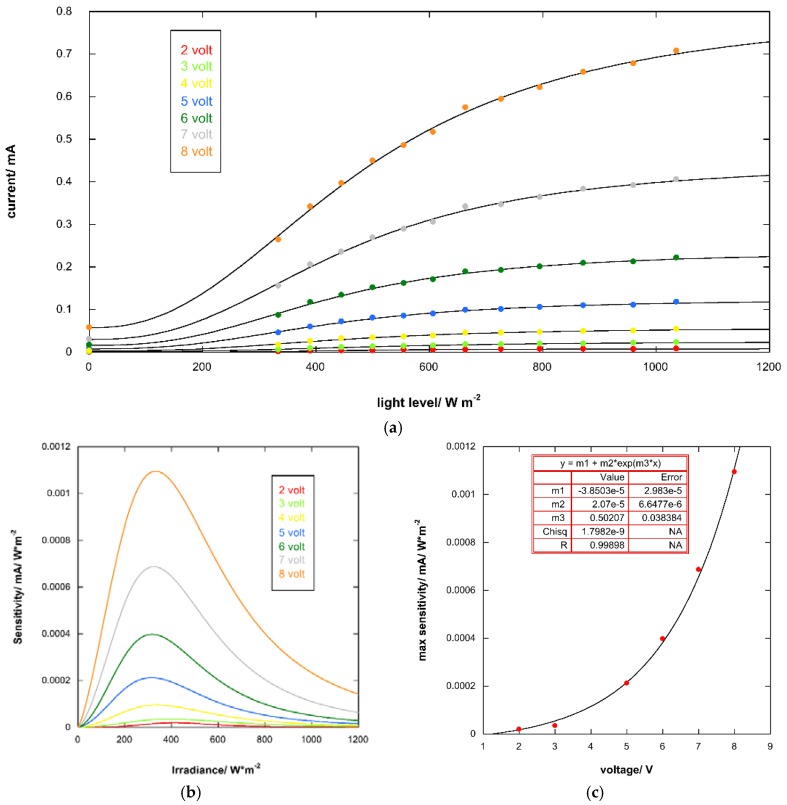
(**a**) Fit of current of photoresponsive device (*i*) vs. irradiance (ϕ) with a sigmoidal function; (**b**) Trend of sensitivity vs. irradiance; (**c**) Max sensitivity vs. voltage.

The I-V characteristics of the photoresponsive device under simulated solar light under different irradiation levels (range 334–1036 W m^−2^) and in dark are reported in Figure 6. The increasing irradiance is clearly associated to a corresponding increase in current. The photoresponsive device sensitivity, *s*, at different potential was evaluated by empirically fitting the current (*i*) vs. irradiance (ϕ) curve with a sigmoidal function (See Figure 7a)

(1) $$i\left(V=const.\right)=a+\frac{b-a}{1+{\left(\frac{\phi }{c}\right)}^{d}}$$

And then

(2) $$\;s={\left(\frac{\partial i}{\partial \phi }\right)}_{V}=-\frac{\left(b-a\right)d{\left(\frac{\phi }{c}\right)}^{d}}{\phi {\left[{\left(\frac{\phi }{c}\right)}^{d}+1\right]}^{2}}$$

where *a*, *b*, *c*, and *d* are constants. The values of sensitivity *vs* irradiance are reported in Figure 7b. The max sensitivity *vs* voltage shows an exponential growth

(3) $$\;s_{max}=\alpha +\beta e^{\gamma V}$$

where α, β, and γ are constants (See Figure 7c).

### 3.5. Response of the Photoresponsive Device

**Figure 8:**
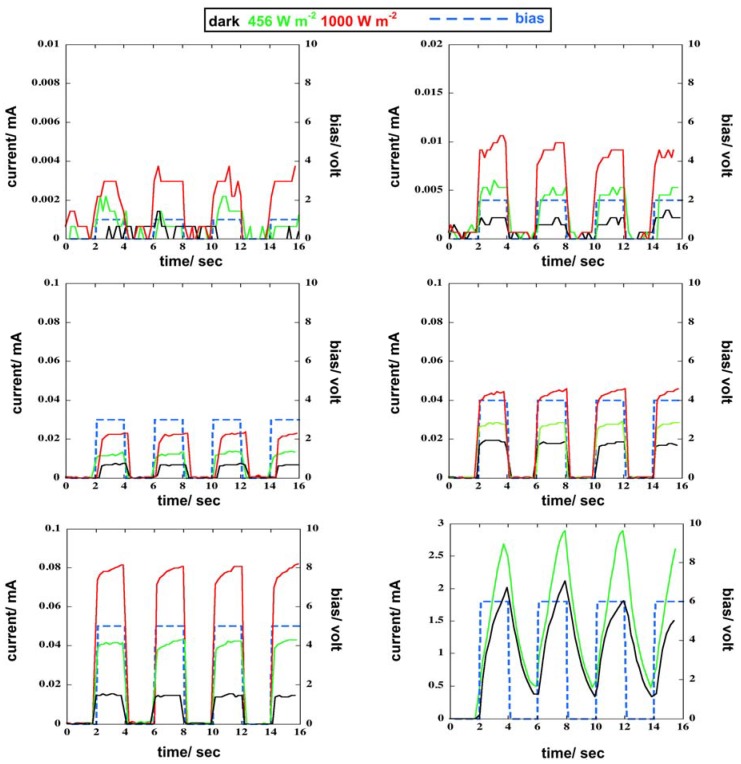
The response of the current of the photoresponsive device vs. forward-biased voltage pulse.

**Figure 9:**
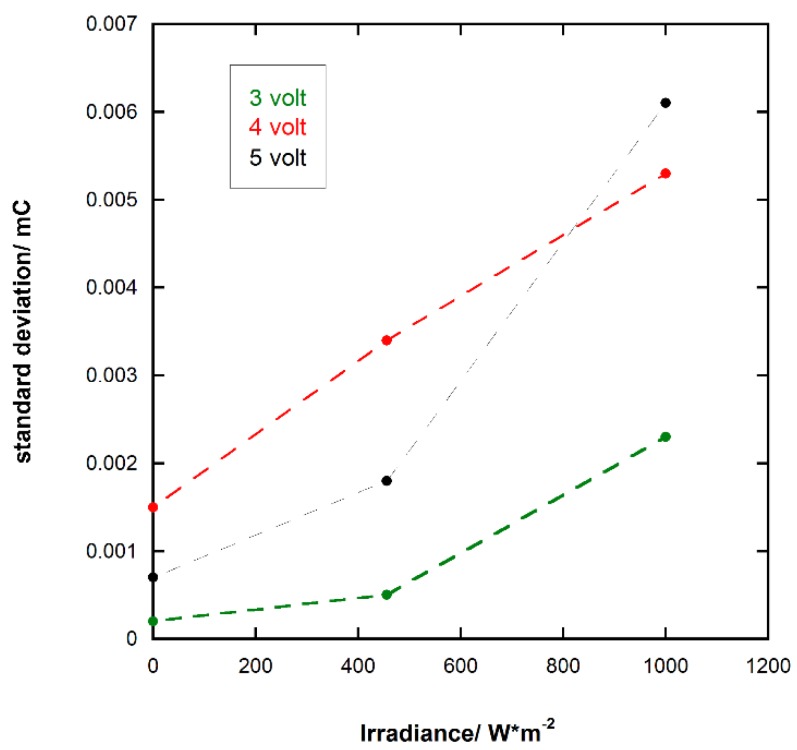
Standard deviation, σ, of the generated charge in photoresponsive device, *q*, vs. voltage pulses.

**Figure 10:**
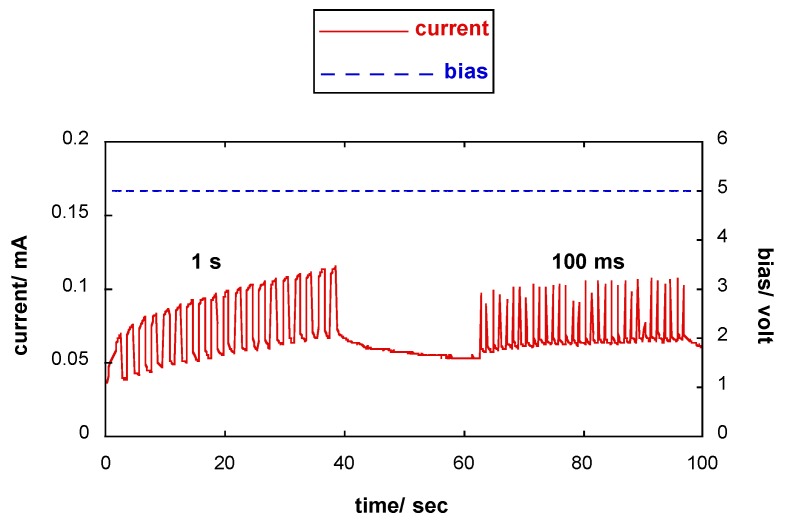

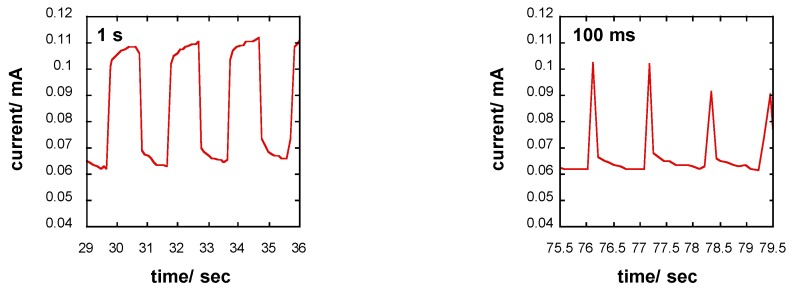
The response of the photodiode to light pulses with a forward bias voltage of 5 V.

The current response of the photoresponsive device to different forward-biased voltage pulses is shown in Figure 8. The best device responses were achieved at voltage values in the 3–5 volt range as shown by the shape of the current pulses. Additionally, to evaluate the reproducibility of the current pulses produced by the device in forward-biased voltage pulse mode, the standard deviation, σ, of the generated charge, *q*, was calculated (see Table 2). The lowest values of σ were achieved for a voltage of 3 V. In addition, for each applied voltage pulse, the reproducibility decreased because the irradiance increased, as shown in Figure 9. Subsequently, using a 5 V forward bias, the response of the photoresponsive device to light pulses was investigated (see Figure 10). Within our instrumental limits, the obtained results show that the device is clearly responsive up to 100 ms light pulses.

### 3.6. J-V Curve of Photoelectrochemical Cell

**Figure 11:**
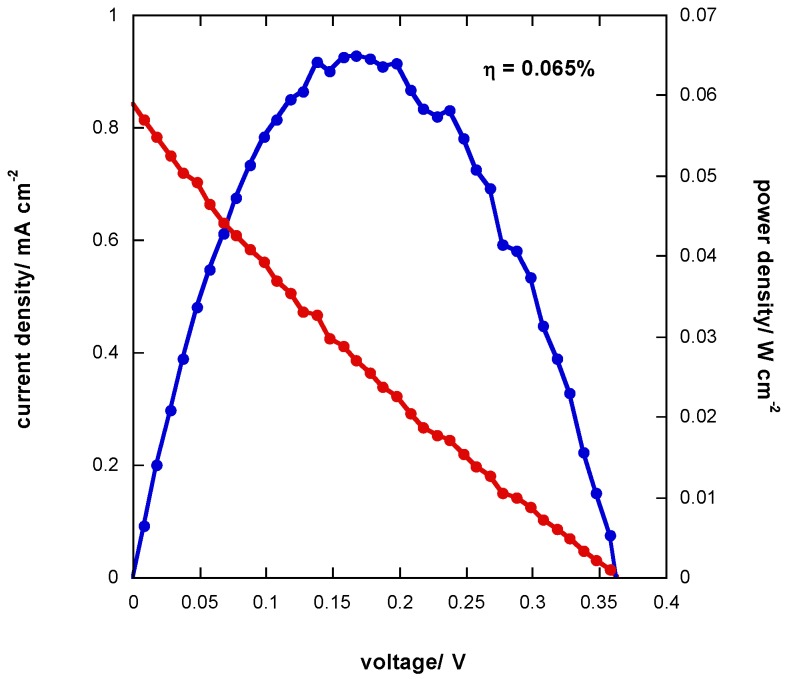
J-V curve (**red line**) and power density (**blue line**) of photoelectrochemical cell.

In Figure 11, the J-V curve and the power density vs. voltage for the assembled photoelectrochemical cell are reported. The open circuit voltage (*V_OC_*) and the short circuit photocurrent (J_SC_) are 0.36 V and 0.8 mA cm^−2^, respectively. The conversion efficiency (*η*) is low, at 0.065%. Notably, the trend of current density vs voltage is linear, thus indicating a very low shunt resistance (*R_sh_*) and a high series resistance (*R_s_*), about 3000 Ω and 5600 Ω, respectively. These values were obtained as follows:

(4) $$\;R_{sh}=-{\left({\frac{dI}{dV}|}_{V=0}\right)}^{-1}$$

(5) $$\;R_{s}=-{\left({\frac{dI}{dV}|}_{V=V_{OC}}\right)}^{-1}$$

The value of *R_s_* is in line with the results obtained by electrochemical impedance spectroscopy analysis of the photoelectrochemical cell (see Section 3.5).

### 3.7. Electrochemical Impedance Spectroscopy (EIS) of Photoelectrochemical Cel

**Figure 12:**
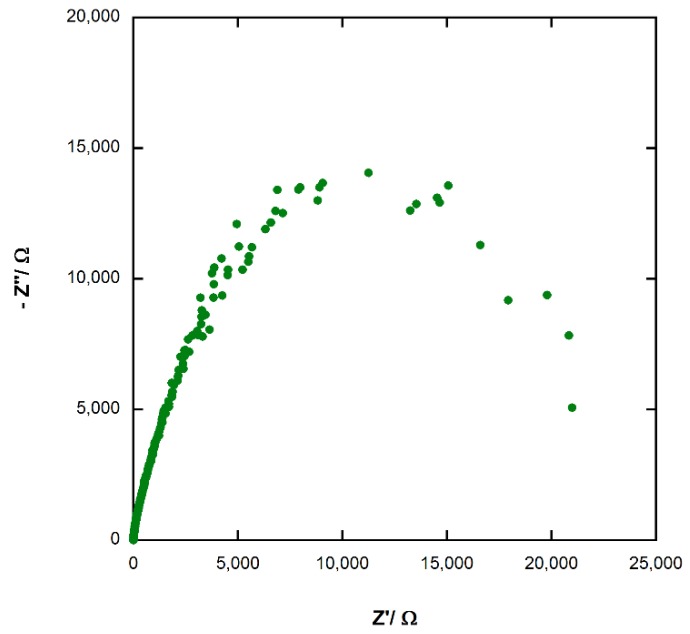
Nyquist plot of photoelectrochemical cell.

The Nyquist plot of the photoelectrochemical cell (see Figure 12) shows a high resistance, with an approximate value of around 11,000 ÷ 13,000 Ω, estimated by the semicircle radius. This result is in total agreement with the low value of conversion efficiency.

### 3.8. Incident Photon to Current Efficiency (IPCE) of Photoelectrochemical Cell

**Figure 13:**
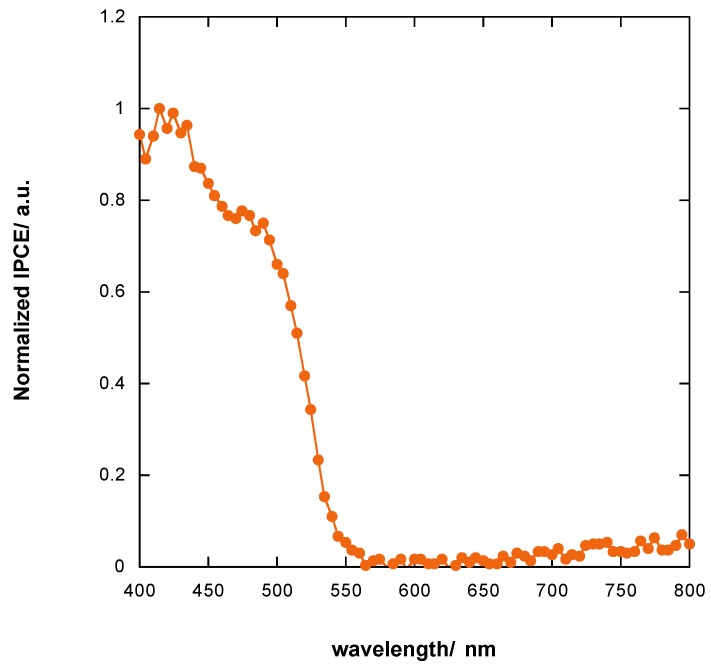
IPCE curve of photoelectrochemical cell.

The normalized quantum efficiency curves of the device are reported in Figure 13. The IPCE curve is fully consistent with the of Pb_3_O_4_ band gap value. Notably, non-zero values of IPCE were obtained for wavelengths shorter than 562 nm, which corresponds to 2.2 eV.

## 4. Conclusions

In this work, a simple and effective procedure for screen printing Pb_3_O_4_ films is proposed. Photoresponsive devices as well as photoelectrochemical cells were produced using these films. Photoresponsive devices based on 3D heterojunction with CuSCN can be easily produced. Taking into account the results obtained in an assessment of the device sensitivity and its responses, the best performances were achieved with a forward bias of 5 V for irradiance levels in the range 200–400 W m^−2^.

With regard to the photoelectrochemical cell, the low recombination (shunt) resistance accounts for its limited photoconversion efficiency in consideration of the J-V curve, which shows a high *R_s_* and a small shunt R_sh_. Therefore, it is reasonable to infer that such behavior of the device is due to the high resistivity of the semiconductor Pb_3_O_4_ [4]. A possible improvement of the efficiency may be achieved by doping the semiconductor with an aliovalent element (e.g., bismuth), which possesses an atomic radius very close to that of lead, thus decreasing the material’s resistivity. On the other hand, electron recombination from Pb_3_O_4_ to electrolyte may be inhibited by replacing the redox couple I^−^/I_3_^−^ with a more suitable one.

## Figures and Tables

**Table 1:** Band gap values of the Pb_3_O_4_ obtained from the absorption onset of the UV-Vis spectra (first line) and those calculated by Tauc’s plots considering the possible transition types. The calculated value that agree better with the absorption onsets is reported in bold.

Band Gap	eV
E_g_ Absorption Onset	2.138 ± 0.004
E_g_ Allowed Direct	2.221 ± 0.001
E_g_ Forbidden Direct	2.162 ± 0.002
E_g_ Allowed Indirect	**2.134 ± 0.004**
E_g_ Forbidden Indirect	2.087 ± 0.006

**Table 2:** Generated charge, *q*, as a result of voltage pulse on three consecutive pulses at different irradiance conditions, their average values, and standard deviations.

Voltage/Volt	Irradiance/W m^−2^	*q*_1_/mC	*q*_2_/mC	*q*_3_/mC	Average Value/mC	Standard Deviation/mC
3	dark	0.0132	0.0135	0.0134	0.0134	0.0002
3	456	0.0252	0.0253	0.0261	0.0256	0.0005
3	1000	0.0440	0.0486	0.0464	0.0463	0.0023
4	dark	0.0393	0.0387	0.0364	0.0381	0.0015
4	456	0.0590	0.0526	0.0575	0.0564	0.0034
4	1000	0.0863	0.0953	0.0958	0.0925	0.0053
5	dark	0.0300	0.0287	0.0298	0.0295	0.0007
5	456	0.0841	0.0876	0.0857	0.0858	0.0018
5	1000	0.1685	0.1574	0.1586	0.1615	0.0061
